# How online shopping festival atmosphere promotes consumer participation in China

**DOI:** 10.1186/s40691-022-00325-5

**Published:** 2023-02-05

**Authors:** Jiali Xie, Namhee Yoon, Ho Jung Choo

**Affiliations:** 1 Department of Textiles, Merchandising, and Fashion Design, Seoul National University, 1 Gwanak-ro, Gwanak-gu, Seoul, 08826 Republic of Korea; 2 Human Ecology Research Center, Korea University, 145 Anam-ro, Seongbuk-gu, Seoul, 02841 Republic of Korea

**Keywords:** Online shopping festival, Online shopping atmosphere, Excitement, Time pressure, Promotional atmosphere, Entertaining atmosphere, Social interaction atmosphere, S–O–R theory

## Abstract

Despite the immense success of China’s *Double Eleven* online shopping festival (OSF), research on how OSFs’ unique atmosphere relates to customer behavior has been scarce. This study investigates the influence of the OSF atmosphere on consumers’ participation behavior based on the stimulus–organism–response framework. Based on the data from 239 young Chinese consumers (in their 20 s and 30 s) and using structural equation modeling, this study explores the influence of three OSF atmosphere types—promotional, entertaining, and social interaction—on consumers’ continuous participation intention. All three types are found to influence consumers’ excitement, which strengthens their continuous participation intention. Additionally, the moderating effect of perceived time pressure is the highest for the relationship between entertaining atmosphere and excitement, followed by that between social interaction atmosphere and excitement. The greater the time pressure perceived by consumers, the stronger is the relationship between entertaining/social interaction atmosphere and excitement. This study bridges the gaps in the literature on atmosphere, consumer sentiment, and festival consumption.

## Introduction

Online shopping festivals (OSFs), which are large-scale commodity promotion events hosted by e-commerce platforms and merchants on specific occasions, are a notable feature of Chinese online marketplace. The *Double Eleven* global OSF was first launched by the Alibaba Group on November 11, 2009, subsequently creating a precedent for e-commerce festival promotions. With a daily turnover of commodities far exceeding expectations, the day “11/11” became a symbolic day for holding large-scale promotional activities and soon became a global shopping carnival day. The gross merchandise volume during OSFs has grown staggeringly every year since 2009 (Wu et al., 2016). The huge daily sales driven by this event now even surpassed those during the traditional “Black Friday” shopping carnival in the west (Chen & Li, 2019). Therefore, it is considered to be the largest and most influential online promotion event in the world (Xu et al., 2017). The success of this OSF indicates the success of its marketing strategies, which have become significant for the development of shopping festivals in other markets. This study, thus, focuses on the *Double Eleven* OSF and explores its influence on consumers.

An OSF is defined as a consumption mode of aggregate promotion (Lu & Zhuang, 2018) that involves marketing activities using various stimuli to attract high customer traffic. Early OSFs were merely driven by the need to satisfy consumers’ utilitarian needs through high discounts such as 50% off promotions. Over time, however, creating a festive atmosphere online has been emphasized to provide consumers with a better shopping experience. Hence, during an OSF, in addition to large-scale (discount) promotions launched within a limited timeframe to stimulate consumers’ participation, entertainment activities such as online games, live streaming, and even competitions are newly introduced features. By playing games on shopping platforms and participating in live shows, consumers can accumulate and redeem coupons and gift cards, which can be used to offset product prices during an OSF. Moreover, universal participation through social networks further influences consumer behavior. Strong social interactions are formed by consumers sharing OSF information as well as their purchasing decisions and participation experience with others. Thus, OSFs have developed from simple price promotion events to a marketing campaigns focusing on creating an entertaining and interactive atmosphere; this indicates the need to explore OSFs’ atmosphere and investigate its impact.

Existing research on shopping atmosphere has focused on its influence in traditional offline (Ha & Lennon, 2011; Kerfoot et al., 2003) and online (Dailey, 2004; Demangeot & Broderick, 2010; Ranganathan, 2012) channels rather than in specific contexts such as OSFs. Thus, there is limited research on the specific classification and definition of OSF atmosphere. Furthermore, research on OSFs is not systematic, as previous studies have merely regarded such festivals as promotion events and focused on their promotion strategies (Akram et al., 2017; Liu et al., 2018). However, recent research has suggested the impact of other aspects of OSFs such as entertainment (Xu et al., 2020) and the social information exchange (Chen & Li, 2020; Li et al., 2020; Xu et al., 2020) on OSF participants’ behaviors. Further, even though OSF activities are being upgraded every year, research on these new features is scarce. Some scholars claim that pleasure and arousal are sufficient to represent the emotions affected by environmental stimuli under pleasure–arousal–dominance theory; they neglect the fact that individuals are often in a complex state of enthusiastic excitement while shopping, especially in the OSF context. Moreover, importantly, OSF events are time-bound; however, the influence of this time limitation on participants has received little empirical attention.

Considering these gaps, this study investigates the influence of the OSF atmosphere on consumers’ excitement and continuous participation intention. It also explores whether or how its characteristic time-limited promotions strengthen OSF atmosphere’s relationship with consumers’ emotions. This study is based on stimulus–organism–response (S–O–R) theory, which states that environmental stimuli affect individuals’ emotions and behaviors (Donovan & Rossiter, 1982; Eroglu et al., 2001; Mehrabian & Russell, 1974). In particular, OSF atmosphere is proposed as an environmental stimulus correlated with consumers’ psychological reactions and behaviors. Thus, by employing S–O–R theory, the current research explores the influence of OSF atmosphere on consumers’ continuous participation intention through their emotions using an online survey of Chinese consumers. A clear understanding of this atmosphere through the S–O–R mechanism could help OSF marketers develop effective marketing strategies and provide suggestions for the sustainable development of shopping festivals.

## Literature review

### Conceptual framework

#### The stimulus–organism–response theory

Mehrabian and Russell’s (1974) S–O–R theory proposes that environmental stimuli (S) affect the internal emotional evaluation of an organism (O), which in turn affects its response (R). Donovan and Rossiter (1982) first applied the S–O–R paradigm to study store atmosphere. They empirically tested their research model using atmospheric cues as environmental stimuli, consumers’ cognitive or emotional perception states as organisms, and approach or avoidance as responses. Eroglu et al. (2001) adopted the S–O–R framework to study the relationship between online store atmosphere and consumer responses. They proposed that in online stores, atmospheric cues (both high and low task-related cues) influence shoppers’ emotional (e.g., happiness) and cognitive states (beliefs, attitudes, perceptions) and subsequently influence their responses. Moreover, numerous research findings support the fact that online store atmosphere affects consumers’ behavior (Wu et al., 2014). In particular, online store atmosphere attributes influence consumers’ emotional states, causing them to alter their purchasing decisions (Lyu et al., 2022) such as prompting consumers to make impulse purchases (Lin et al., 2022). OSF is a unique context of online shopping that creates an atmosphere that can arouse positive emotions and stimulate consumption among consumers (Xu et al., 2020). OSF participants’ decision-making processes are more likely to be guided by emotional states during OSFs than in regular online shopping settings, wherein customers pay more attention to identifying product features and quality. Therefore, by considering the S–O–R model, this study proposes that OSF atmosphere (as stimulus) affects participants’ emotional reactions (as organisms), which in turn affects their continuous participation intention (as responses).

#### OSF atmosphere

Kotler (1973) proposed the concept of atmosphere, which represents the quality of a market’s surrounding space and can positively influence consumer behavior by evoking pleasant feelings. Research on shopping atmosphere includes studies of the atmosphere in physical stores and during online shopping. First, store atmosphere is defined as the physical surroundings consciously designed to affect shoppers’ responses, such as their perception of the store’s sensory qualities, emotional state, and purchase likelihood (Kotler, 1973). A good physical store atmosphere can attract more visitors and influence their satisfaction and purchase decisions (Ha & Lennon, 2011; Kerfoot et al., 2003). Second, online atmosphere is the conscious design of online spaces to ensure they positively impact users (Dailey, 2004). Similar to the stimuli in bricks-and-mortar stores, online atmospheric cues can provide information about retailers, such as their type and quality level, and in turn influence consumers’ responses. Demangeot and Broderick (2010) claimed that customers are perceptive about the online shopping environment, which can help build their shopping interest. Moreover, researchers have found considerable evidence supporting the impact of online atmosphere on consumer behavior (Ranganathan, 2012). Atmosphere-related stimuli in online stores, such as music, pictures, and product descriptions, are important factors that affect browsers’ pleasant feelings, attitudes toward online stores, and purchase decisions (Eroglu et al., 2003; Mummalaneni, 2005). Richard (2005) classified online atmosphere into high and low task-related atmosphere types that each influence consumers’ purchasing behavior. High task-related atmosphere provides cues to help shoppers achieve their shopping goals, such as information on price promotions, shopping policies, and product images. Low task-related atmosphere includes information indirectly related to shopping goals, such as fonts, colors, and sounds. Entertainment or recreation activities provide low task-related atmosphere cues, as they create a fun, imaginative, and exciting online environment (Richard et al., 2010).

An OSF is a unique online shopping context reflected in the shopping festival atmosphere it creates (Xu et al., 2020). An OSF atmosphere requires platforms and retailers to consciously design the festival environment to elicit positive emotions among customers, thereby stimulating their willingness to buy. Chen and Li (2019) explained the OSF atmosphere perceived by consumers through economy, festival entertainment, and mass participation. OSF atmosphere contain specifically designed elements including large-scale promotions, entertainment, and interactive activities. Based on an analysis of the online atmosphere of OSFs, this study categorizes OSF atmosphere into three types: promotional atmosphere, entertaining atmosphere, and social interaction atmosphere.

First, discount promotions are an essential feature of OSFs. Promotions are a common marketing method under which a product’s price is temporarily reduced or the quantity of the product at a certain unit price is increased (Raghubir & Corfman, 1999). Introducing short-term incentives such as promotions can stimulate a strong and immediate customer purchase response (Kotler, 1973). Compared with daily promotions, promotions during OSFs include larger price discounts, more diverse forms of entertaining and interactive activities through which consumers can earn shopping allowances, and a more limited time and quantity. Hence, retailers create a promotional atmosphere by offering price discounts, vouchers, coupons, shopping allowances, monetary incentives, gift cards, and platform-based activities.

Second, the variety of entertainment activities during OSFs further stimulates consumers’ continuous participation intention. Through mini-games, participants can browse product and store information and obtain shopping vouchers that could incentivize further shopping, thereby raising their purchase intention for certain products. The game reward amount can be increased by inviting friends. Therefore, OSF games and online carnival parties create an entertaining atmosphere for consumers.

Third, with the development of social platforms and interactive technologies, OSFs make online shopping even more interactive. Fan et al. (2019) and Park et al. (2014) found a positive relationship between online interactions and consumers’ online purchases. Given the mass participation in OSFs, the media and other people influence participants, and their emotions and behaviors may change accordingly (Chen & Li, 2020). Hence, this study defines social interaction atmosphere as that formed by the dissemination and communication of OSF-related information by consumers. During an OSF, consumers form an OSF community through social media and their social behavior can influence their consumption behavior. When a community has frequent social interactions, an effective social interaction atmosphere can be generated.

#### Excitement

Excitement is a feeling of enthusiasm and desire that motivates people to act (Ahn & Shin, 2015). In the field of marketing, excitement is conceptualized as an emotional response, defined as a combination of high pleasure and high arousal (Baker & Wakefield, 2012; Liljander & Bergenwall, 1999). According to the S–O–R framework (Mehrabian & Russell, 1974), environmental stimuli affect consumer sentiment, such as pleasure and arousal, which act as mediating variables in determining consumer response behavior. Previous studies have investigated the link between online atmosphere and consumption-based emotional responses; they found that elements of online atmosphere have a spontaneous and significant effect on consumer sentiment (Davis et al., 2008; Floh & Madlberger, 2013). Kim and Lennon (2010) and Loureiro et al. (2013) supported this view and showed that a website’s visual elements such as its display, layout, color, and links increase buyers’ emotions of pleasure and arousal; these represent the major emotions displayed in response to environmental stimuli (Eroglu et al., 2003; Russell, 1979). In addition, arousal (or high activation) and pleasure (or positive valence) can be interpreted as expressions of excitement (Russell & Barrett, 1999; Tsai et al., 2018). Therefore, in this study, excitement is defined as the degree of pleasure and arousal that consumers perceive while participating in OSF activities. This research considers excitement as consumers’ emotional reaction and proposes that OSF atmosphere has a positive impact on the participant’s excitement. Thus, the following hypotheses are proposed.

- *Hypothesis 1 *(*H1*): OSF atmosphere positively influences excitement.
- *H1a*: OSFs’ promotional atmosphere positively influences excitement.
- *H1b*: OSFs’ entertaining atmosphere positively influences excitement.
- *H1c*: OSFs’ social interaction atmosphere positively influences excitement.

#### Continuous participation intention

According to the S–O–R framework, affective factors elicit responses, which translate into behavioral intentions or actual behaviors. In the context of OSFs, this study considers continuous participation intention as consumers’ willingness to participate in an OSF in the future, including their willingness to share information, engage in entertainment activities, play games, browse, search, and make purchases. Continuous participation intention is correlated with consumers’ previous experiences of the same activities, and individuals with this intent are more likely to spread positive word-of-mouth about festival events (Choo et al., 2016).

Previous research has found that atmospheric cues influence consumers’ affective responses and, subsequently, their actual responses. Pleasant emotional responses elicited by online atmosphere influence consumers’ approach behaviors, such as their engagement, participation levels, and purchase intentions (Mazaheri et al., 2011), increasing their willingness to revisit (Davis et al., 2008; Eroglu et al., 2003). Thus, in the following hypothesis, a positive relationship is expected between excitement and continuous participation intention.

- *H2*: Excitement positively influences continuous participation intention in the context of OSFs.

#### Perceived time pressure

As a type of stress, time pressure is a situational variable that influences consumers’ decision-making while shopping (Duncan Herrington & Capella, 1995; Iyer, 1989; Vermeir & Van Kenhove, 2005). Howard and Sheth (1969) defined time pressure as the inverse of the time required to execute a purchase action. It is the perceived need or desire to complete a task quickly (Carnevale & Lawler, 1986). Thus, perceived time pressure is defined as the scarcity of time available to make decisions compared with the time needed (Liu et al., 2017). Moreover, in the field of psychology, time pressure is known to induce emotion-based decision-making (Maule et al., 2000; Shiv & Fedorikhin, 2002). Previous research has shown that time pressure intensifies consumers’ arousal (Jones et al., 2011) and may increase their emotional impulse purchases (Sohn & Lee, 2017). It can also cause pleasurable stress, known as eustress, which is a positive psychological response (Maule et al., 2000).

**Fig. 1:**
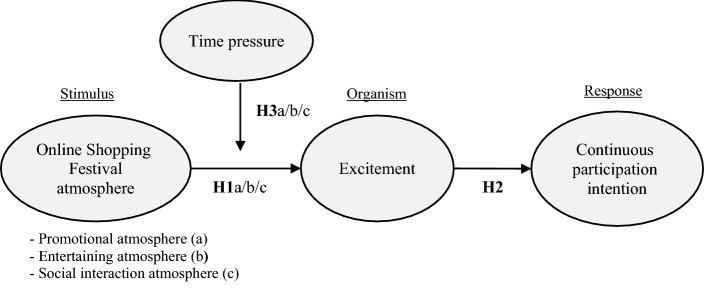
Conceptual framework

In the context of this research, the relationship between OSF atmosphere and excitement may vary by situation, signifying the need to examine moderating variables. OSFs’ promotional, entertainment, and interactive activities are time-limited, and consumers’ perceptions of lack of time can affect their emotional responses. Atmosphere’s influence on emotion can, thus, become stronger when consumers perceive time pressure. Therefore, this study defines perceived time pressure as consumers’ perceived sense of time urgency while participating in OSFs and proposes that it moderates the relationship between OSF atmosphere and excitement. The research framework is shown in Fig. 1.

- *H3*: The more the consumers perceive time pressure, the stronger is the relationship between OSF atmosphere and excitement.
- *H3a*: The more the consumers perceive time pressure, the stronger is the relationship between promotional atmosphere and excitement.
- *H3b*: The more the consumers perceive time pressure, the stronger is the relationship between entertaining atmosphere and excitement.
- *H3c*: The more the consumers perceive time pressure, the stronger is the relationship between social interaction atmosphere and excitement.

## Methods

### Measurements

This study used six constructs: promotional atmosphere, entertaining atmosphere, social interaction atmosphere, excitement, continuous participation intention, and perceived time pressure, whose measures were adopted from previous studies. The items for promotional (two items), entertaining (three items), and social interaction atmosphere (three items) were adopted from Yu et al. (2018), Chen and Li (2020), and Li et al. (2020), respectively. Excitement was measured using four items with two subdimensions, namely, pleasure (two items) and arousal (two items), adopted from Xu et al., (2017, 2020). The measures of continuous participation intention (two items) were adopted from Li et al. (2020). Table 2 presents the wording of the aforementioned measures. Further, perceived time pressure was measured using three items, adopted from Gelbrich and Sattler (2014): *I find myself pressed for time when I do my festival shopping*; *I am in a hurry when I do my festival shopping*; and *I have only a limited amount of time available to do my festival shopping.* All the items used in this study were modified to fit the OSF setting and measured using a five-point Likert scale (1= “strongly disagree” to 5= “strongly agree”).

Consumers’ OSF experience was evaluated using the period of OSF participation and OSF shopping behaviors. The time period of OSF participation was measured by the question, “*when did you start participating in OSFs such as Double Eleven?*” Questions on OSF shopping behaviors were related to the money spent and types of products purchased during the 2021 OSF. The original measurements in English were translated into Chinese, and bilingual native Chinese researchers read the survey repeatedly to ensure the sentences were natural and clearly conveyed the original meaning.

### Data collection and sample

Data were collected through an online survey using a convenience sample from China. The questionnaire link was created on the professional online survey site, *wjx.cn*, and shared widely using social media platforms such as WeChat. After obtaining informed consent from participants, screening questions such as whether they were Chinese nationals, lived in China, and had participated in the 2021 *Double Eleven* OSF were asked at the start of the survey to identify participants’ eligibility. Those who responded with a “yes” could proceed to the rest of the survey. Therefore, the sample only included Chinese consumers living in China who had participated in the most recent OSF. Eligible participants were then asked to recall their experience of participating in the 2021 OSF and answer the survey, which was divided into three parts. The first part contained questions on respondents’ OSF experience. The second part measured the study variables. The final section collected demographic information, including gender, age, education, occupation, and income.

The investigation period was from November 27 to December 27, 2021, and each participant was paid 1 USD for completing the survey. A total of 239 valid responses were collected. The sample consisted of 148 female (61.9%) and 91 male (38.1%) respondents, of which 87.9% were in their 20s and 12.1% were in their 30s. Data collection was conducted without age screening, and respondents consisted of Chinese consumers in their 20s and 30s who had participated in the OSFs. According to Alibaba Group (2021), young consumers are the main participants in the *Double Eleven* OSF in China. Most respondents had a college education (82.0%) and were unmarried (90.0%). Of all the respondents, 64.9% were students and 24.2% were employed. The data were collected from different regions of China and were geographically extensive. Further, most of the participants lived in major cities of China, including Hangzhou (31.4%), Shaoxing (5.9%), Shanghai (5.4%), Guangzhou (5.4%), and Beijing (4.2%). This trend was consistent with that of online shopping consumption, reflecting the representativeness of the sample. The respondents reported shopping online 6.74 times per month on average and that they had *Double Eleven* OSF participation experiences of 5 years on average. During the 2021 *Double Eleven* OSF, the respondents shopped the most on Taobao (97.9%), Tmall (3.8%), JD.COM (3.8%), Pinduoduo (2.5%), and other platforms (2.1%). They spent an average of CNY 4238 (approximately USD 665) on these online shopping platforms, and 75.3% of them bought clothes. Detailed sample characteristics are shown in Table 1.

**Table 1:** Sample characteristics

Variable		Percentage (%)
Gender	Female	61.9
	Male	38.1
Age (in years)	20–29	87.9
	30–39	12.1
Occupation	Student	64.9
	Employee	24.2
	Other	10.9
Residential city in China	Hangzhou	31.4
	Shaoxing	5.9
	Shanghai	5.4
	Guangzhou	5.4
	Beijing	4.2
	Other	47.7
OSF participation experiences (*Double Eleven* OSF engagement period of years)	Fewer than 3 years	23.4
	3–5 years	36.0
	More than 5 years	40.6
Average amount spent on online shopping platforms during the 2021 *Double Eleven* OSF	Fewer than CNY 1000	30.5
	CNY 1000–3000	37.3
	CNY 3000–5000	13.4
	More than CNY 5000	18.8
Products purchased during the 2021 *Double Eleven* OSF (purchase rate of each item)	Clothing	75.3
	Accessories	18.8
	Shoes and hats	39.3
	Bags	10.5
	Cosmetics	36.4
	Food	51.5
	Daily necessities	72.8
	Electronics	28.5
	Others	8.4

Note. OSF = online shopping festival

## Results

### Measurement validity

The validity and reliability of all study variables except for perceived time pressure were tested through confirmatory factor analysis using AMOS 23.0. Owing to the sensitivity of chi-square to the sample size, normed chi-square, goodness-of-fit index (GFI), comparative fit index (CFI), root mean square residual (RMR), and root mean square error of approximation (RMSEA) were evaluated as fit indices. Following Hair et al. (2010), Table 2 shows an acceptable fit with the data (*χ*^2^ = 111.714, *df* = 55, *p* < 0*.*001; normed *χ*^2^ = 2.031; GFI = 0*.*935; CFI = 0*.*969; RMR = 0*.*037; RMSEA = 0*.*066). All the factor loadings were statistically significant at *p* < 0*.*01 and larger than the 0.60 threshold (Bagozzi & Yi, 1988). The average variance extracted (AVE) for the constructs was higher than 0.60 (Bagozzi & Yi, 1988), and the composite reliability (CR) of the constructs ranged from 0.781 to 0.895 (> 0.70 threshold; Nunnally & Bernstein, 1994); therefore, convergent validity was satisfactory. The reliability of the measurements was tested using Cronbach’s alpha. The Cronbach’s alpha values ranged from 0.766 to 0.884 (> 0.70 threshold; Nunnally & Bernstein, 1994), which indicated satisfactory internal consistency for all the scales. The measurement of perceived time pressure also was reliable (Cronbach’s alpha = 0.898).

**Table 2:** Confirmatory factor analysis results

Construct	Items	Standardized loading (λ)^a^	Cronbach’s α	AVE	CR	Mean
Promotional atmosphere	I saved money through discounts available during the online shopping festival (OSF)	0.898	0.766	0.643	0.813	3.791
	I think the OSF offers me quality products at competitive prices	0.693				
Entertaining atmosphere	The OSF’s promotion was creative and interesting	0.866	0.877	0.707	0.844	3.273
	The OSF’s promotion activities were full of variety and fun	0.835				
	The OSF’s promotion activities (e.g., live shows and games) were interactive and entertaining	0.821				
Social interaction atmosphere	I communicated with my friends or family about my experience during the OSF to socialize	0.868	0.779	0.648	0.781	3.659
	I enjoyed socializing with others when I find a good deal in the OSF	0.737				
Excitement	I felt energized to use the OSF platform	0.860	0.884	0.658	0.895	3.640
	I felt enthusiastic while engaging in the OSF platform	0.856				
	I felt that the OSF can gratify my shopping needs with fun	0.786				
	I felt happy when participating in the OSF activities with my friends	0.735				
Continuous participation intention	I prefer participating in the next OSF compared to other people	0.853	0.817	0.692	0.801	3.389
	I will prepare well in advance before the next OSF	0.810				

^a^All estimates are statistically significant at *p* < 0.01

To test the discriminant validity, the squared correlations of the constructs and AVE values were compared for each pair of constructs (see Table 3). All the squared correlations were lower than the corresponding AVEs (Fornell & Larcker, 1981). Thus, the discriminant validity of the constructs was acceptable.

**Table 3:** AVE and squared correlation

	Promotional atmosphere	Entertaining atmosphere	Social interaction atmosphere	Excitement	Continuous participation intention
Promotional	0.643^a^				
Entertaining	0.338^b^	0.707			
Social interaction	0.196	0.293	0.648		
Excitement	0.393	0.591	0.288	0.658	
Participation	0.484	0.538	0.278	0.630	0.692

^a^Average variance extracted (AVE) values for the constructs are displayed on the diagonal

^b^Numbers below the diagonal are the squared correlation estimates of the two variables

### Hypothesis testing

#### Structural model

AMOS 23.0 was used to perform structural equation modeling to test the hypotheses. The fit of the structural model was satisfactory (*χ*^2^ = 135.587, *df* = 58, normed *χ*^2^ = 2.338). GFI (0.922) and CFI (0.958) values (> the 0.90 threshold) as well as RMR (0.047) and RMSEA (0.075) (< the 0.80 threshold) all indicated an acceptable fit (Hair et al., 2010).

**Fig. 2:**
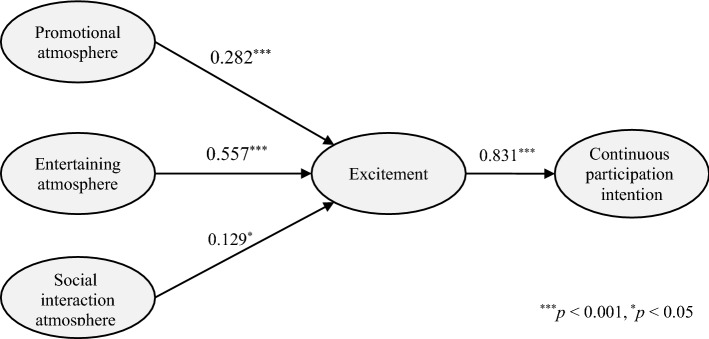
Structure model. *χ*^2^ = 135.587 (*df* = 58, *p* < 0.001), normed *χ*^2^ = 2.338, GFI = 0.922, CFI = 0.958, RMR = 0.047, RMSEA = 0.075

The results showed that all three OSF atmosphere types had significant relationships with excitement. Figure 2 shows that entertaining atmosphere had the greatest statistically significant influence on excitement (γ = 0.557, *p* < 0.001), followed by promotional atmosphere (γ = 0.282, *p* < 0.001) and social interaction atmosphere (γ = 0.129, *p* < 0.05). In turn, excitement significantly influenced continuous participation intention (β = 0.831, *p* < 0.001). Thus, H1a, H1b, H1c, and H2 were supported. Additionally, the indirect effects of OSF atmosphere on continuous participation intention were tested through bias-corrected bootstrapping with 5000 samples on AMOS. The indirect effects of promotional atmosphere (Effect = 0.234, 95% confidence interval (CI) [0.134, 0.558]), entertaining atmosphere (Effect = 0.413, 95% CI [0.294, 0.554]), and social interaction atmosphere (Effect = 0.124, 95% CI [0.033, 0.270]) on continuous participation intention were statistically significant.

#### Moderating effect of perceived time pressure

To test H3, which is about the moderating effect of perceived time pressure for the relationships between the three atmosphere variables and excitement, we conducted bias-corrected bootstrapping using Hayes’ (2018) Process Macro Model 1 with 5000 samples. The CI was set to 95%. Each of the OSF atmosphere types was used separately as an independent variable. Online shopping frequency (i.e., average online shopping frequency per month), and expenditure during the OSF (i.e., amount spent on an online platform during the 2021 *Double Eleven* OSF) were entered as the control variables.

According to the bootstrapping results for testing H3a, the interplay effect of promotional atmosphere and perceived time pressure on excitement was statistically insignificant (*b* = 0.062, 95% CI [− 0.044, 0.169]). Thus, H3a was rejected.

The results of the bootstrapping analysis via the Process Macro Model 1 similarly showed the moderating effects of perceived time pressure on the relationship between entertaining atmosphere and excitement (*b* = 0.067, 95% CI [0.002, 0.133]) and between social interaction atmosphere and excitement (*b* = 0.085, 95% CI [0.004, 0.170]). As shown in Table 4, at all perceived time-pressure levels, OSF atmosphere significantly influenced excitement. Further, when consumers perceived high time pressure (Mean + 1 *SD*), the relationship between entertaining atmosphere and excitement was the strongest (Effect = 0.549, 95% CI [0.442, 0.653]). The relationship between entertaining atmosphere and excitement increased with an increase in consumers’ perceived time pressure. As shown in Table 5, when a consumer perceived high time pressure (Mean + 1 *SD*), social interaction atmosphere had the greatest impact on excitement (Effect = 0.461, 95% CI [0.331, 0.608]). The results imply that the more consumers perceive time pressure while shopping during OSFs, the stronger is the relationship between entertainment/social interaction atmosphere and excitement. Thus, H3b and H3c were supported.

**Table 4:** Conditional effects of entertaining atmosphere on excitement at different levels of perceived time pressure

Perceived time pressure	Effect	Boot*SE*	95% Confidence Interval	
			LLCI	ULCI
Mean − 1 *SD* (2.133)	0.376	0.061	0.254	0.497
Mean (3.333)	0.457	0.039	0.378	0.536
Mean + 1 *SD* (4.666)	0.549	0.052	0.442	0.653

*LLCI* lower bound confidence interval, *ULCI* upper bound confidence interval

**Table 5:** Conditional effects of social interaction atmosphere on excitement at different levels of perceived time pressure

Perceived time pressure	Effect	Boot*SE*	95% confidence interval	
			LLCI	ULCI
Mean − 1*SD* (2.133)	0.245	0.070	0.107	0.383
Mean (3.333)	0.347	0.047	0.253	0.441
Mean + 1*SD* (4.666)	0.461	0.074	0.313	0.608

*LLCI* lower bound confidence interval, *ULCI* upper bound confidence interval

## Conclusions

### General discussion

OSFs such as the *Double Eleven*, *Double Twelve*, *Jingdong 618*, and *Black Friday* have become major marketing events for e-commerce platforms. Just as *Black Friday* is viewed as a shopping ritual for preparing Christmas gifts in the west, China’s OSFs have also become a ritual for young Chinese shoppers. The online atmosphere created by e-commerce platforms has produced significant effects despite the virtual nature of the online shopping environment, including consumers’ inability to directly touch goods, unlike in a physical store. Compared with regular promotion events, OSFs have a unique atmosphere that plays a key role in consumers’ purchase decisions. However, OSFs’ atmosphere must be able to stimulate consumers’ consumption activities to gain the expected results during the festival’s promotional period. Consumers differentiate their expectations from OSFs and other repetitive events hosted during specific seasons from their everyday shopping expectations. Thus, OSFs’ unique atmosphere should aim to create a shopping ritual to increase the time consumers spend on shopping platforms and increase purchase intentions. The success of OSFs demonstrates the experience of online festivals’ atmosphere marketing, which provides a unique background for this research.

The current study utilized the S–O–R framework to empirically test whether stimuli (OSF atmosphere) influence consumers’ internal states (emotions), and in turn lead to behavioral responses (continuous participation intention). Three atmosphere types—promotional, entertaining, and social interaction—were proposed, and their influence on consumers’ continuous participation intention by building excitement was explored. In addition, the moderating effect of perceived time pressure on the relationship between OSF atmosphere and excitement was examined. The results indicate that, first, all three proposed OSF atmosphere types have a positive and significant relationship with excitement, with entertaining atmosphere having the greatest impact. Second, excitement strengthens consumers’ continuous participation intention. The more excited consumers are, the stronger is their intention to participate in the OSF again. Third, consumers’ perceived time pressure during OSFs strengthens the relationships between entertaining/social interaction atmosphere and excitement. These findings are consistent with those of previous studies, which show that shoppers’ perceptions of the environment elicit positive emotions, which in turn influence their approach behavior (Donovan & Rossiter, 1982; Eroglu et al., 2001). The empirical study by Grewal et al. (2003) showed that consumers’ intention to visit a store is highly influenced by their perceptions of its atmosphere and that consumers display stronger emotional arousal when perceiving time pressure.

The influence of the three OSF atmosphere types proposed in this study is also supported by evidence from existing research. OSFs differ from normal online shopping environments (Xu et al., 2020) and use innovative promotion strategies besides product promotions, such as creating an entertaining atmosphere using the Internet and social media. First, regarding promotional atmosphere, the success of the *Double Eleven* OSF mainly stems from its successful online promotion strategies (Akram et al., 2017; Liu et al., 2018). This OSF stimulates mass participation and raises consumers’ enthusiasm by gathering retailers and products and launching large-scale, limited period promotions (Lu & Zhuang, 2018; Wu et al., 2016). Second, regarding entertaining atmosphere, the findings of Chen and Li (2019) confirmed that consumers’ perceived levels of festival entertainment positively influence their purchase intention during an OSF. Further, Xu et al. (2020) claimed that gamification is a determinant of consumers’ use of shopping platforms. Third, several researchers confirm that social interaction can stimulate consumption. Consumers are more likely to buy more, spend more, and make impulse purchases when they are influenced by their social networks (Li et al., 2020; Luo, 2005). Similarly, social interaction has been proven to be an important factor influencing consumers’ participation intentions in OSFs (Xu et al., 2020). To obtain various rewards and discounts during OSFs, consumers share information about the festival on social networks and invite others to participate, thus forming strong interactions with family and friends. In conclusion, the promotional, entertaining, and social interaction atmosphere types significantly and positively relate to individuals’ emotional and behavioral responses during OSFs.

### Theoretical implications

This study makes several contributions to the literature. First, this is the first empirical study to extend the concept of atmosphere from the perspective of shopping festivals to the specific context of OSFs by classifying OSF atmosphere into three prominent types. Most existing studies on shopping atmosphere only cover day-to-day online shopping. Although some studies on OSFs have touched upon the concept of atmosphere, they take the perspective of consumer perception. Chen and Li (2019) conceptualized promotion strategies from the perspective of consumers, who are tempted by three main drivers: price promotions, the fun of promotional activities, and the contagiousness of mass participation. Li et al. (2020) discussed consumers’ perceptions of fairness atmospherics. Moreover, other studies on OSFs are not about atmosphere. They explore the mechanisms of the relationship between socioeconomic or psychological factors and specific OSF consumer behaviors, such as information incentives and social influence (Xu et al., 2017), promotion size and social interaction (Li et al., 2020), and utilitarian and hedonic motivations (Akram et al., 2017; Xu et al., 2020). Therefore, the current study contributes theoretically by proposing the promotional, entertaining, and social interaction atmosphere types of OSFs, filling a gap in existing research.

Second, no published research has adopted excitement to measure the emotional state of consumers during OSFs. The present research suggests excitement as a mediator in the relationship between OSF atmosphere and consumer behavioral intention, thus expanding the literature on consumer sentiment. Although numerous studies have adopted pleasure, arousal, and dominance as consumers’ affective response states, many researchers believe that pleasure and arousal are sufficient to represent the range of emotions evoked by environmental stimuli (Eroglu et al., 2003; Russell, 1979). Accordingly, even in OSF research, scholars have mostly employed pleasure and arousal to measure consumers’ emotional perceptions (Xu et al., 2020). Furthermore, excitement is conceptualized as an affective state achieved by the combination of pleasure and arousal (Baker & Wakefield, 2012). Studies indicate that excitement plays an essential role in people’s consumption experience (Lesser & Kamal, 1991). This research is, therefore, the first to comprehensively assess excitement as a positive psychological state, demonstrating the mechanism between OSF atmosphere and intended behavior.

Third, perceived time pressure, another important feature of OSFs, has received limited empirical attention. OSFs involve limited period promotions held on specific festival days set by e-commerce companies. Thus, consumers can only participate in promotional activities and enjoy special offers when they purchase within the limited timeframe set by the retailer or platform. Thus, unlike previous studies that have neglected the influence of stress on OSF participants, this study investigated the moderating effect of perceived time pressure on the relationship between OSF atmosphere and excitement.

The results of this study have both similarities with and differences from the findings of previous research on OSFs. One consistent finding is that utilitarian and hedonic OSF atmospheric cues perceived by consumers during a festival, positively impact consumers. Consumers’ perceived price value has a significant positive effect on their emotions (pleasure and arousal), and perceived gamification has a significant effect on arousal (Xu et al., 2020). In addition, Chen and Li (2020) demonstrated that economic, festival entertainment, and mass participation factors positively impact consumers’ participation intentions. Among these, consumer-perceived economic factors were found to have the greatest impact on participation intention, followed by mass participation and festival entertainment factors (Chen & Li, 2020). However, in this study, entertaining atmosphere had the strongest influence on participants’ emotions (excitement), followed by promotional atmosphere and social interaction atmosphere. This may be because this study explores the influence of OSF atmosphere on continuous participation intention through emotions, while Chen and Li (2020) investigated the direct effect of OSF stimuli on participation intention. Differences in survey periods may be another explanation, as this study investigates the influence of the 2021 *Double Eleven* OSF, while previous studies were mostly conducted before 2020 and OSFs’ strategies have evolved since the COVID-19 pandemic. Further, activities based on large-scale promotions to create festive entertainment are gaining increased attention. The empirical results of this study, thus, provide theoretical support for the influence of OSF atmosphere since the pandemic.

### Managerial implications

The insights from the study’s findings have important managerial implications as well. Initially, the study verified that atmosphere, which is time-consuming and costly to build, affects consumers’ emotions and behaviors during OSFs. This finding has practical implications for e-commerce platforms striving to create a festive, immersive, and entertaining atmosphere for consumers. It also highlights the need to formulate marketing strategies that excite consumers and increase their continuous participation intention. Nowadays, OSFs have become essential marketing events and their influence is growing globally. Hence, this research provides a real-world guide for retailers that want to imitate OSFs or create their own unique atmosphere.

This research also provides practical suggestions to improve e-commerce platforms’ atmosphere and promote sales during large-scale promotions. First, retailers and e-commerce platforms are recommended to create diverse types of shopping atmosphere, such that they have a positive impact on consumer sentiment and behavior. The empirical results show that the promotional atmosphere type, as a utilitarian dimension, and the entertaining and social interaction atmosphere types, as hedonic dimensions, all raise participants’ excitement levels, thereby increasing their intention to participate in OSFs in the future. In the case of the *Double Eleven* OSF, Alibaba has been successful at grabbing consumers’ attention. To convey information about the OSF to potential participants, Alibaba employed various strategies such as large sales discounts, used incentives to motivate participants to socially interact through the mass media and social media, and invited celebrities to participate in TV shows, live performances, and live streaming. In addition, to enhance participants’ entertainment experience during the OSFs, Alibaba designed various fun and rewarding games and activities to incentivize the consumers. As this study shows that online shopping atmosphere has a positive impact on consumer sentiment, e-commerce platforms should focus on atmosphere building to enhance their competitive advantages.

Second, focusing on the emotional needs of consumers and increasing their excitement levels is an effective marketing strategy for e-commerce platforms organizing OSFs. Our results show that when participating in an OSF, consumers experience a complex sense of excitement, rather than singular emotions such as pleasure or arousal. Stimulated by the shopping atmosphere, consumers’ purchase intention rises and excitement plays a mediating role in this. The more excited consumers are, the more willing they are to participate in the OSF in the future. However, different atmosphere types impact consumers’ excitement differently, with entertaining atmosphere having the greatest influence, followed by promotional atmosphere and social interaction atmosphere. Based on this finding, e-commerce platforms can strive to improve their OSF atmosphere based on their shortcomings as well as their marketing activities. In the future, brands can use technology to create a more entertaining and interactive atmosphere during OSF promotions, thereby enhancing the exciting experience of participants and promoting consumer engagement. Technologies such as augmented reality, virtual reality, and artificial intelligence can be employed to enhance the presence of and pleasure from OSF entertainment activities. Platforms could also adopt metaverse technology to build OSF virtual reality worlds and enhance consumers’ interaction experiences during OSFs. Furthermore, they could display the remaining time prominently and emphasize the countdown to increase consumers’ likelihood of perceiving time pressure. Our results show that a high degree of perceived time pressure strengthens the relationships between a festival’s entertaining/social interaction atmosphere and excitement. For example, retailers can increase the impact of perceived time pressure by, for example, offering special promotional prices or gifts for the first few orders to stimulate consumer demand.

In conclusion, this study provides theoretical and practical guidance for retailers, brands, and e-commerce platforms globally to improve their OSF marketing strategies and increase consumers’ willingness to participate in OSFs. Other countries can also draw lessons from China’s successful experience when developing their own OSF campaign. For example, top marketplaces such as Lazada and Shopee have launched *Double Eleven* OSF in Southeast Asia to boost consumer engagement and purchase intention (Handayani et al., 2022).

### Limitations and future studies

This study has some limitations. First, it was conducted in China, where the *Double Eleven* OSF originated. The collected data were from Chinese participants and, therefore, the findings cannot be generalized to the global market. Future research is required to investigate the impact of OSFs in other cultural contexts. Second, this study only targeted young consumers in China; future research could include a wider and more diverse sample. In addition, this study only proposed three OSF atmosphere types and explored their positive relations; their potential negative relations, such as consumers’ negative emotional response, could be discussed in future research along with the inclusion of more OSF atmosphere types. Moreover, whether the impact of OSFs differs by country, cultural background, age, gender, income, or education level, should also be explored in the future to enable e-commerce platforms to formulate more specific and targeted marketing strategies. Further, the measures applied to the OSF context were adopted from the literature; however, some items are potentially double-barreled and may have potential validity concerns. Future research could develop concrete measurements based on this study’s conceptual variables to ensure they have satisfactory reliability and validity.

## Data Availability

The datasets used and/or analyzed during the current study are available from the corresponding author on reasonable request.
